# Late follicular progesterone to estradiol ratio is not influenced by protocols or gonadotropins used

**DOI:** 10.1186/s12958-015-0116-y

**Published:** 2015-11-05

**Authors:** E. Shalom-Paz, N. Aslih, N. Samara, M. Michaeli, A. Ellenbogen

**Affiliations:** Department of Obstetrics and Gynecology, IVF unit, Hillel Yaffe Medical Center, Faculty of Medicine, Technion – Israel Institute of Technology, Hadera, Israel

**Keywords:** Progesterone, Progesterone/estrogen ratio, Pregnancy rate, Delivery rare, ROC (AUC)

## Abstract

**Objective:**

Increased progesterone level during follicular phase seemed to be associated with decreased pregnancy rate.

**Study design and methods:**

A prospective cohort study, 1.1.2012 - 31.8.13. The Progesterone (P) and Progesterone/Estrogen (P/E2) level on ovulation induction day were compared between the protocols and the different gonadotropins used. Roc analysis was calculated to determine the cutoff of P/E2 to predict delivery rates. P/E2 ratio was calculated as PX1000/e2 level.

**Main results:**

One hundred thirty-nine patients were enrolled to the study. No difference in the P level at hCG stimulation day between different protocols, however, E2 and P/E2 ratio were significantly lower in the long protocol compare with antagonist protocol 1757.7 ± 923.2 vs. 1342.9 ± 1223; *P* = 0.003 and 0.48 ± 0.31 vs. 0.83 ± 0.87; *P* = 0.038). The endometrium was significantly thicker in the long group compare with short and antagonist. Significantly more top-quality embryos (TOP) were achieved in the antagonist group. Comparable results between the types of gonadotropins used in regards with cycle characteristics and pregnancy and delivery rates. The P/E2 ratio which can predict live birth rate was found to be 0.45, AUC = 0.632, *p* = 0.02 and 95 % CI 0.525–0.738 and a significantly higher pregnancy and delivery rates at a P/E2 bellow 0.45.

**Conclusion:**

Endometrial receptivity is determined by the complex interactions of E2 and P.

## Capsule

More Pregnancies and deliveries after IVF cycles were achieved in women who had lower progesterone to estradiol ratio on day of hCG.

## Introduction

Progesterone plays an important role during luteal phase, particularly vital in creating decidualization changes needed for implantation and progression of pregnancy. In IVF treatments premature progesterone elevation occurs between 5 and 30 % of the treatments despite the use of GnRH analogs [1–3]. The increased progesterone level during follicular phase seemed to be associated with decreased pregnancy rate [4, 5].

The pathophysiology which creates the premature elevation of progesterone is not clear; some suggested it follows an increase number of follicles and in the presence of high estradiol levels [5]. The correlation between progesterone and estradiol was evaluated in several studies in different population. Younis et al. claimed that P/E2 ratio was more accurate to detect low ovarian reserve and less oocyte were retrieved on OPU [6]. Other studies showed that elevation in P/E2 ratio revealed higher oocytes collection and didn’t harm the pregnancy rates in normo-ovulatory patients [1, 7, 8].

To date, the approach to patients with elevated P levels on the hCG administration day is under a controversy whether embryos should be transfer or not. On one hand, the raised peripheral concentrations of progesterone in the late follicular phase are likely to influence the secretory changes of the endometrium, leading to impaired endometrial receptivity and premature decidualization [9] and as a consequence a decreased probability for ongoing pregnancy [1, 2, 4]. On the other hand the oocyte and embryo quality were not adversely affected [10, 11].

To the best of our knowledge, no data was published comparing the difference between treatment protocols and type of gonadotropins administered during stimulation and progesterone levels progesterone/estradiol ratio and treatment outcome.

We assumed that the GnRH analog and gonadotropins used during stimulation may have an influence on progesterone level and P/E2 ratio. The primary objective was to establish a serum P and P/E2 ratio threshold that would define detrimental levels to cycle outcomes. As a secondary objective, we aimed to determine the correlation between serum P levels and P/E2 ratio on the day of hCG administration to the different stimulation protocols, gonadotropins used and pregnancy and delivery rates.

## Material and methods

### Study participants

Patients undergoing fresh ART were enrolled prospectively to participate in the study between 1.1.2012 and 31.8.13 at the IVF unit in Hillel Yaffe Medical Center. To reflect the broad range of patients typically encountered in clinical practice, no inclusion/exclusion criteria were applied regarding baseline characteristics except women’s age <42. Institutional review board approval was obtained and all patients signed an informed consent in order to participate in the study.

### Treatment protocol

The treatment protocol, type and doses of gonadotropins were prescribed on a case-by-case basis according to patient characteristics and clinician preferences and judgment. The initial dose of gonadotropin was individualized for each patient according to age, basal FSH levels, antral follicle count, body mass index (BMI), and previous response to ovarian stimulation. Dose adjustments were performed according to ovarian response, which was monitored by vaginal scans and estradiol determinations. Three main protocols were included in the study: long agonist protocol, short flare protocol and antagonist protocol. All treatments protocols were conducted as previously described [12–14].

### Medication used

Patients underwent controlled ovarian stimulation by one of four possible methods: recombinant follicle stimulating hormone (rFSH) alone (Gonal-F, Merck-Serono; or Puregon, MSD); highly purified human menopausal gonadotropin (HPhMG) alone (Menopur, Ferring Pharmaceutical); or rFSH combined with HP-hMG.

### Hormone level follow-up

Estrogen and P levels were routinely performed during the study on every follow-up visit, including on the day of hCG (Ovitrelle Merck-Serono) administration before egg retrieval. The P/E2 ratio was calculated as [P (ng/mL) * 1000/E2 (pg/mL)] [15, 16].

After oocyte retrieval, in vitro fertilization (IVF) or intracytoplasmic sperm injection (ICSI) was performed. The quality of all available embryos was evaluated, and up to two embryos were transferred on day 2 or 3 of development. Embryo quality was evaluated on the day of transfer according to cells number, symmetry, granularity, type, percentage of fragmentation, presence of multinucleate blastomers, and degree of compaction as previously described [17]. A top-quality embryo was described as an embryo with 4–5 cells on day 2 or on day 3, >6 cells equally sized blastomers and < =20 % fragmentation and no multinucleate cells.

### Pregnancy determination

The β-hCG test was performed 14 days after embryo transfer, and the clinical pregnancy and implantation rate was confirmed when a gestational sac with fetal heart beat was visible by ultrasound examination after 7 weeks of pregnancy.

Demographic data, treatment information and results and pregnancy outcome were recorded and followed until delivery.

Power analysis was conducted to answer the main study’s questions pregnancy rate in low vs. high progesterone level and P/E2 ratio above and below 0.48. It was calculated that 70 women in each group were needed to detect of 80 % probability power of 22 % difference in live birth rate (20 and 42 %) between different values of progesterone and P/E2 ratio based on Cetinkaya et al. [18] who demonstrated a threshold of P/E2 of 0.48 and progesterone level of 1.5 ng/ml at a significance level (alpha) of 0.05.

### Statistical analysis

Statistical analysis was performed using the SPSS software package version 21 (SPSS Inc., Chicago, IL). We used Shapiro Wilks test to evaluate the distribution of the quantitative parameters in the data. Comparisons between groups were analyzed using Student’s *t* test or Mann–Whitney *U* test Anova and Kruskal Wallis when appropriate. Proportions were compared using Chi Square test or Fisher exact test. P value of less than 0.05 was considered significant. We used a multivariate logistic regression analysis model to rule out any other confounders that can influence the clinical results. A receiver operating characteristics (ROC) analysis was performed to determine the most efficient cut-off values for the P/E2 ratio to discriminate between successful and unsuccessful IVF/ICSI-ET outcomes. The highest value of the area under the curve (AUC) was determined.

## Results

One hundred thirty nine patients were enrolled in the study. The main characteristics of the group including the cause of infertility and treatment protocols are presented in Table 1.

**Table 1:** The influence of 3 different protocols on treatment’s parameters

	Long-Ago (*N* = 55)	Short-Ago (*N* = 25)	Antagonist (*N* = 59)	p
Age	30.87 ± 4.94	37.00 ± 5.40	32.44 ± 5.50	^#^0.0001
				^@^0.002
Cause of Infertility				
Mechanical	3 (5.5 %)	4 (16.0 %)	8 (13.8 %)	
PCO	4 (7.3 %)	0	4 (6.9 %)	
Male	26 (47.3 %)	6 (24 %)	20 (34.5 %)	
Combined	4 (7.3 %)	5 (20 %)	6 (10.3 %)	
Unexplained	10 (18.2 %)	6 (24 %)	13 (22.4 %)	
low ovarian reserve	1 (1.8 %)	2 (8 %)	2 (3.4 %)	
endometriosis	7 (12.7 %)	0	3 (5.2 %)	
Other	0	2 (8 %)	2 (3.4 %)	
E2 (pmol/dl)	1757.7 ± 923.2	1498 ± 763	1342.9 ± 1223	*0.003
P (ng/mL)	0.6787 ± 0.3169	0.7424 ± 0.3619	0.6925 ± 0.3336	0.66
P/E_2_ ratio	0.48 ± 0.31	0.63 ± 0.54	0.83 ± 0.87	*0.038
Endometrial thickness (mm)	10.8 ± 2.3	8.7 ± 2.1	9.5 ± 2.2	*0.031
				^#^0.003
Number of M2 oocytes	8.4 ± 4.5	5.0 ± 3.0	5.9 ± 4.9	*0.003
				^#^0.007
Number of 2PN	5.8 ± 3.1	3.7 ± 2.2	4.5 ± 3.4	*0.049
				^#^0.03
Fertilization rate	69.4 ± 22.7	77.6 ± 20.05	80.7 ± 18.9	*0.025
Cleavage rate	97.51 ± 10.13	100 ± 0	98.51 ± 6.3	0.23
Number of Embryos transfer	2.05 ± 0.48	2.2 ± 0.77	1.88 ± 0.53	0.064
Treatment outcome				
Chemical pregnancy (%)	22 (40 %)	6 (24 %)	22 (37 %)	0.37
Clinical pregnancy (%)	19 (34.5 %)	6 (24 %)	19 (32 %)	0.64
Life birth rate (%)	17 (31 %)	4 (16 %)	14 (24 %)	0.34
TOP Quality embryos (%)	42 (76 %)	22 (88 %)	56 (95 %)	*0.006

Values are mean ± SD, n, or n/total (%). * Statistical differences between Long vs. Antagonist # Statistical differences between Long vs. Short @ Statistical differences between Antagonist vs. Short

Comparing the 3 different protocols we could not find any difference in the P level on hCG stimulation day, however, estradiol and P/E2 ratio were significantly lower in the long agonist protocol compare with antagonist protocol 1757.7 ± 923.2 vs. 1342.9 ± 1223; *P* = 0.003 and 0.48 ± 0.31 vs. 0.83 ± 0.87; *P* = 0.038). Concomitantly, the endometrium was significantly thicker in the long agonist group compare with short and antagonist. On the other hand, significantly more TOP quality embryos were achieved in the antagonist group (Table 1).

Comparing the IVF treatment outcome in regards the gonadotropins used we could not demonstrate any significant difference between the r-FSH and HP (hMG) used in the different protocols of treatment beside embryo’s quality (Table 2).

**Table 2:** Comparable results in different gonadotropins used for stimulation

	r-FSH (*N* = 77)	hMG (*N* = 45)	Combined Gonadotropins (*N* = 17)	p
E2 (pmol/dl)	1467.2 ± 886.2	1461.6 ± 823.4	2036.0 ± 1892.6	0.45
P (ng/mL)	0.71 ± 0.34	0.63 ± 0.32	0.77 ± 0.28	0.28
P/E_2_ ratio	0.73 ± 0.79	0.54 ± 0.42	0.59 ± 0.42	0.33
Endometrial thickness (mm)	9.9 ± 2.3	9.6 ± 2.5	10.2 ± 2.2	0.68
Number of M2 oocytes	7.2 ± 4.4	5.4 ± 3.4	8.9 ± 6.8	0.05
Number of 2PN	5.2 ± 3.2	3.9 ± 2.6	5.8 ± 4.5	0.057
Fertilization rate	77.04 ± 20.2	75.02 ± 22.08	70.7 ± 23.8	0.63
Cleavage rate	98.4 ± 5.4	99.26 ± 4.9	95.7 ± 13.31	0.27
Number of Embryos transfer	1.9 ± 0.5	2.1 ± 0.65	2.1 ± 0.64	0.08
Treatment outcome				
Chemical pregnancy (%)	32 (42 %)	12 (27 %)	6 (35 %)	0.25
Clinical pregnancy rate (%)	27 (35 %)	11 (24 %)	6 (35 %)	0.45
Live birth rate (%)	21 (27 %)	9 (20 %)	5 (29 %)	0.61
Quality (1 or 1 + 2)	70 (91 %)	38 (84 %)	12 (71 %)	*0.038 1 vs. 3

Values are mean ± SD, n, or n/total (%). *statistical significant between r-FSH vs. Combined Gonadotropins

**Fig. 1:**
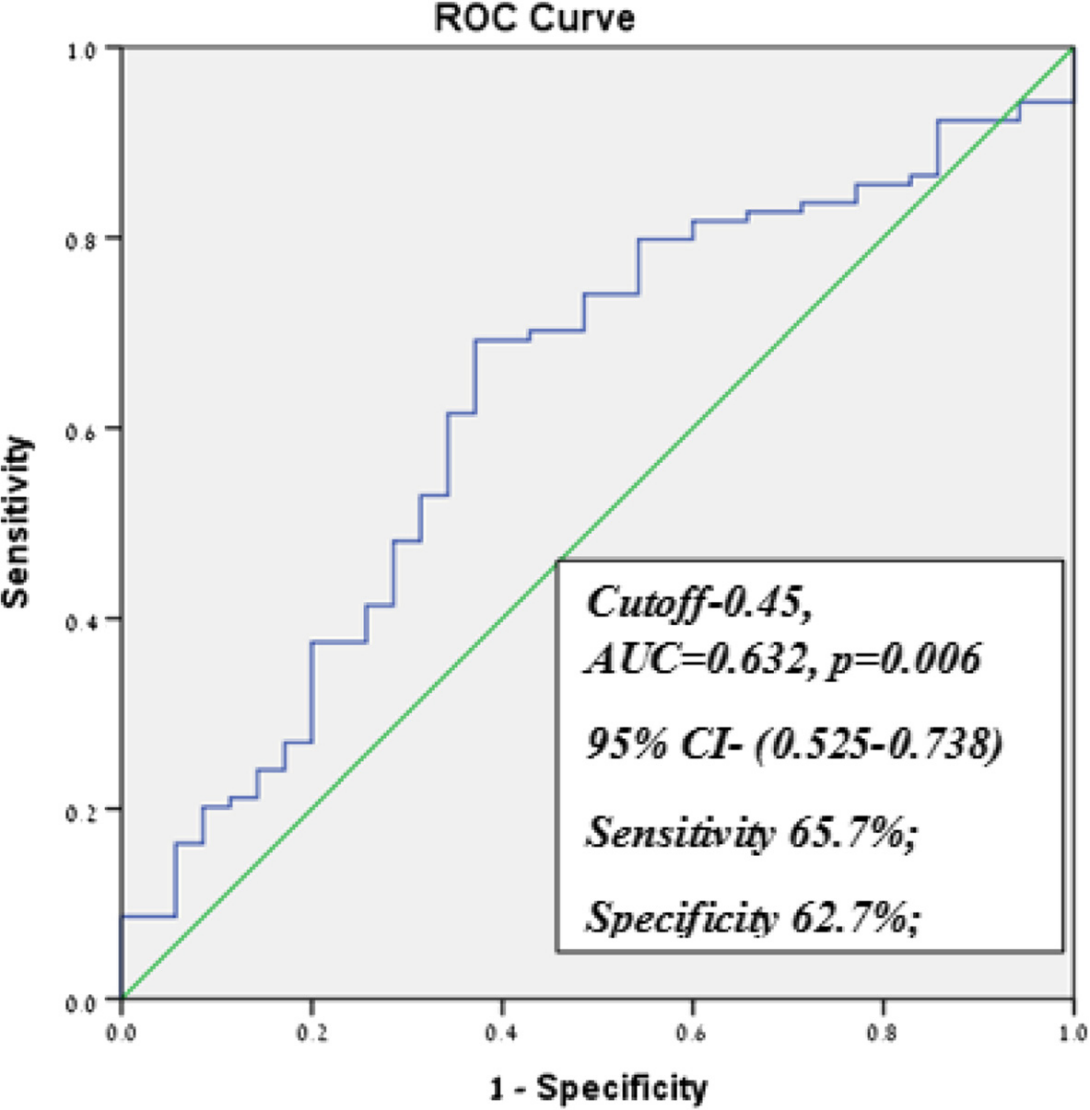
ROC curve for P/E2 ratio to predict live birth rate

According to ROC analysis, the P/E2 ratio which can predict live birth rate was found to be 0.45, AUC = 0.632, *p* = 0.006 and 95 % Confidence Interval 0.525–0.738 with sensitivity of 65.7 %; specificity of 62.7 %; and overall accuracy of 64 % (Fig. 1).

We found that although TOP quality embryos, fertilization rate and cleavage rate were comparable, endometrial thickness, estradiol level on day of hCG, number of M2 oocytes collected, clinical pregnancy rate and most importantly live birth rate were significantly higher when P/E2 ratio was lower than 0.45 group (Table 3).

**Table 3:** Measurements for ratio p/E_2_ ≤0.45

	P/E_2_ ≤0.45	P/E_2_ >0.45	*p*
	(*n* = 63)	(*N* = 76)	
E2 (pmol/dl)	2032.5 ± 1182.3	1122.5 ± 697.7	*P* < 0.001
P (ng/mL)	0.5492 ± 0.2379	0.8177 ± o.3483	*P* < 0.001
Endometrial thickness (mm)	10.3 ± 2.1	9.6 ± 2.5	*P* = 0.037
Number of M2 oocytes	8.62 ± 5.04	5.35 ± 3.64	*P* < 0.001
Number of 2PN	5.98 ± 3.49	3.95 ± 2.82	*P* < 0.001
Fertilization rate	72.6 ± 21.9	78.2 ± 20.38	*P* = 0.15
Cleavage rate	98.7 ± 4.23	98.1 ± 9.6	*P* = 0.60
Number of Embryos transfer	2.11 ± 0.48	1.92 ± 0.63	*P* = 0.033
Treatment outcome			
Chemical pregnancy	27 (42.9 %)	23 (30.3 %)	*P* = 0.16
Clinical pregnancy	25 (39.7 %)	19 (25 %)	*P* = 0.07
Live birth rate	23 (36.5 %)	12 (15.8 %)	*P* = 0.006
TOP quality embryos	56 (88 %)	64 (84 %)	*P* = 0.47

Values are mean ± SD, n, or n/total (%)

In Table 4, comparing the live birth to no deliveries cycles we could show that the only difference was found to be in the P and P/E2 ratio. However in both groups the progesterone level was in normal range.

**Table 4:** Comparison between delivered and non-delivered group

	No live born	Life born	*p*
	*n* = 104	*n* = 35	
Age	33.0 ± 5.9	31.4 ± 4.8	*P* = 0.15
E2 (pmol/dl)	1545.26 ± 1128.4	1504.3 ± 776.49	*P* = 0.64
P (ng/mL)	0.73 ± 0.34	0.57 ± 0.26	*P* = 0.016
P/E_2_ ratio	0.72 ± 0.73	0.47 ± 0.33	*P* = 0.020
Endometrial thickness (mm)	9.86 ± 2.43	9.96 ± 2.08	*P* = 0.80
Number of M2 oocytes	6.66 ± 4.62	7.34 ± 4.64	*P* = 0.39
Number of 2PN	4.65 ± 3.24	5.5 ± 3.4	*P* = 0.17
Fertilization rate	74.28 ± 22.1	79.6 ± 17.9	*P* = 0.28
Cleavage rate	99.1 ± 8.5	99.2 ± 3.84	*P* = 0.49
Number of Embryos transfer	2.01 ± 0.58	2.00 ± 0.54	*P* = 0.76
Protocol			
Long	38 (69 %)	17 (31 %)	
Short	21 (84 %)	4 (16 %)	*P* = 0.34
Antagonist	45 (76.3 %)	14 (23.7 %)	
Total gonadotropin dose median (range)	1500 (130–4000)	1300 (750–3000)	*P* = 0.012

Values are mean ± SD, n, or n/total (%)

We conducted a univariate analysis in order to predict a live birth rate. Taking into consideration P/E2 ratio, endometrial thickness, number of MII oocytes retrieved, type of gonadotrophins used, treatment protocol, total dosage of gonadotrophins and number of 2PN achieved, we found that the total dosage of gonadotrophins and progesterone level on hCG day were significantly correlated with good pregnancy outcome.

Multivariate Logistic regression model was conducted on the basis of the univariate analysis. We included parameters that reduce multi-collinearity, such as age, endometrial thickness, number of MII oocytes retrieved and number of 2PN, P/E2 ratio and P/E2 ratio <0.45. We found that the P/E2 ratio <0.45 increased the chance for live birth with odds ratio of 2.8, *p* = 0.021, 95 % CI = 1.18–7.07.

## Discussion

In this study we evaluated prospectively the association between treatment protocol, gonadotropins used and the level of progesterone and P/E2 ratio on day of hCG administration for ovulation and their impact on treatment outcome and pregnancy and delivery rate. ROC analysis established a cutoff value predicting a threshold which distinguish between live birth and non-live birth cycles. We demonstrated that a P/E2 ratio with a cutoff <0.45 was predictive for live birth with total accuracy of 64 %. In Table 4, comparing the live birth to no deliveries cycles we could show that the only difference was found to be in the P and P/E2 ratio, however in both groups the progesterone level was in normal range, however significantly lower in the group of live birth. Elgindy et al. suggested a cutoff of 0.55 to predict clinical pregnancy in agonist cycles [16]. Others suggested cut-offs of P/E2 <0.48 to achieve higher pregnancy and deliveries rates, which is relatively similar to our result, however they analyzed antagonist cycles only [18]. As far as we know this is the only study that analyze prospectively different protocols and determined a general cutoff regardless the treatment protocol treated.

We found that neither the treatment protocol nor the gonadotropins used had influence on the pregnancy outcome. The Progesterone and Progesterone/Estradiol ratio were statistically lower in the long agonist protocol. However, there was no impact on embryo quality in the antagonist protocol although P/E2 was significantly higher. This finding is in agreement with previous studies which demonstrated that high P/E2 ratio has no effect on oocytes and embryo’s quality [19, 20].

The association between hormone levels and pregnancy results in IVF-ICSI cycles are still investigated as the main focus of the clinical aspect of pregnancy outcome. It is well accepted that one of the rate limiting steps to achieve live birth is the window of implantation. A current view is that the optimal synchronization between decidualization, receptivity and embryo’s invasion are reflected in that particular narrow phase. In IVF-ET cycles, we occasionally cause supra-physiological estrogen and progesterone levels, which are source of variations and alterations for oocytes and embryo quality, endometrial thickness and receptivity, and therefore, impairs pregnancy and delivery rates [21–23].

Contrary to the above said, in the study of Requena et al., analyzed retrospectively 2850 cycles of high responders’ women, they aimed to determine the influence of high progesterone levels on clinical outcomes in the context of high ovarian response. Interestingly, contradicting other studies mentioned above, including our study, they demonstrated that increased progesterone levels was correlated with high estradiol levels and did not have any detrimental effect on cycle outcome [24].

Bourgain et al. describe different luteal proliferative changes comparing stimulated cycles and natural cycles. In the stimulated cycles unfavorable effect was demonstrated during the early luteal phase and they were corrected during the late luteal phase [25]. In agreement, Elgindy et al. in their study they revealed that endometrial receptivity could not be the only mechanism to alter implantation. They describe different implantation rate between cleavage stage embryo transfer and blastocysts transfer. In this study they showed lower implantation rate on day 3 transfer which was completely recovered when embryos were transferred as blastocysts [26]. Probably the adverse effect of P/E2 in stimulated cycles were corrected waiting longer for day 5 transfer and by that overcoming the miss match of receptivity.

In our study we evaluated the correlation of P/E2 with different protocols and we followed the delivery rate, by that we created more generalized information, however this study was not randomized study.

Our conclusion is that P/E2 ratio <0.45 may be a good prognostic factor for delivery rate in the main 3 protocol used in IVF-ICSI cycles. Different gonadotropins used may have no impact on P/E2 ratio and cycle outcome. A large cohort study may lighten the influence of different protocols of stimulation on P/E2 ratio and live birth rate.
